# Prevalence, Clinical Profile, and Outcomes of Ectopic Pregnancy at a Teaching Hospital in a Low‐Resource Setting, Northern Ethiopia: A 5‐Year Retrospective Chart Review

**DOI:** 10.1155/jp/8791113

**Published:** 2026-02-06

**Authors:** Hale Teka, Mohamedawel Mohamedniguss Ebrahim, Mohammedtahir Yahya, Bisrat Tesfay Abera, Ephrem Berhe, Hiluf Ebuy Abraha, Fanos Gebru, Awol Yemane

**Affiliations:** ^1^ Department of Obstetrics and Gynecology, Faculty of Medicine, College of Health Sciences, Mekelle University, Mekelle, Ethiopia, mu.edu.et; ^2^ Faculty of Medicine, College of Health Sciences, Mekelle University, Mekelle, Ethiopia, mu.edu.et; ^3^ Department of Internal Medicine, Faculty of Medicine, College of Health Sciences, Mekelle University, Mekelle, Ethiopia, mu.edu.et; ^4^ Arnold School of Public Health, University of South Carolina, Columbia, USA, sc.edu

**Keywords:** ectopic pregnancy, epidemiology, prevalence, surgical management

## Abstract

**Background:**

Ectopic pregnancy remains a significant cause of maternal morbidity and mortality worldwide, particularly in low‐resource settings. The aim of this study was to investigate the prevalence, clinical profile, and management outcomes of ectopic pregnancies at Ayder Comprehensive Specialized Hospital (ACSH) in Ethiopia between January 1, 2017 and December 31, 2021.

**Methods:**

A cross‐sectional study of 152 women diagnosed with ectopic pregnancy and admitted to ACSH between 2017 and 2021 was conducted. Data, including sociodemographic characteristics, obstetric history, clinical presentation, diagnostic methods, intraoperative findings, and management outcomes, were collected retrospectively from medical records. The prevalence of ectopic pregnancy was calculated based on the total number of deliveries during the study period. Descriptive statistics were used to summarize the data.

**Results:**

Of 23,090 deliveries in the ACSH between 2017 and 2021, 152 cases of ectopic pregnancy were registered, corresponding to a prevalence of 6.58 per 1000 deliveries. The average age of the women was 28 years (SD ± 0.5), with the majority (55.5%) between 25 and 34 years old. Most patients (78.3%) lived in rural areas. Multigravida women accounted for 58.6% of cases. There was a history of abortion in 28.3% of women and a history of previous ectopic pregnancy in 6.6%. The most common clinical findings were tender abdomen (84%), adnexal motion tenderness (53%), and cervical motion tenderness (45%). Hemoglobin levels below 11 mg/dL were observed in 41% of cases. The majority of ectopic pregnancies were diagnosed using both clinical assessment and ultrasound (88.8%). Surgical management was the primary treatment modality (92.1%), with salpingectomy performed in 92.8% of cases. Blood transfusions were required in 29.6% of patients. The median length of hospitalization was 3 days (IQR = 2).

**Conclusions:**

With a prevalence of 6.58 per 1000 deliveries, ectopic pregnancy remains a major health problem in ACSH. Most patients presented with acute symptoms requiring surgical intervention. Early detection and improved access to reproductive health services could reduce the morbidity of ectopic pregnancy in the region.

## 1. Introduction

An ectopic pregnancy is defined as the implantation of a fertilized ovum outside the uterine cavity, usually in the fallopian tubes [1]. It is a life‐threatening condition and a significant cause of maternal morbidity and mortality [2]. Globally, ectopic pregnancies account for approximately 1%–2% of all reported pregnancies [3]. However, the incidence varies due to different risk factors and access to health care in different populations and settings [4].

In high‐income countries, advances in diagnostics, such as transvaginal ultrasonography and sensitive human chorionic gonadotropin (hCG) testing, have facilitated the early detection of ectopic pregnancies [5, 6]. This has led to improved management options, including medical therapy with methotrexate, and reduced morbidity and mortality associated with this condition [7]. Conversely, in low‐income countries, however, limited access to healthcare services, lack of diagnostic facilities, and delayed presentation contribute to higher morbidity and mortality rates [8].

Ectopic pregnancy is still one of the main causes of pregnancy‐related deaths in low‐income countries. In sub‐Saharan Africa, ectopic pregnancy is estimated to be responsible for up to 10% of maternal deaths [9]. The clinical presentation often includes abdominal pain, vaginal bleeding, and amenorrhea, but symptoms can be nonspecific, leading to delays in diagnosis [10]. Complications such as tubal rupture and hemorrhagic shock are more common in low‐resource settings due to delayed diagnosis and treatment [11].

In Ethiopia, the burden of ectopic pregnancy is exacerbated by challenges such as limited access to reproductive health services, low utilization of antenatal care (ANC) and inadequate emergency obstetric care [12]. Cultural factors, socioeconomic barriers, and lack of awareness contribute to delayed healthcare‐seeking behavior among women [13]. Studies have shown that ectopic pregnancies are a significant cause of maternal morbidity and mortality in Ethiopia [14, 15].

Despite their importance, there is limited data on the epidemiology and management outcomes of ectopic pregnancies in Ethiopia, particularly in the Tigray region. Understanding the patterns of ectopic pregnancies, associated risk factors and management outcomes is critical for developing strategies to improve maternal health services. The aim of this study was to assess the prevalence, clinical presentation and management outcomes of ectopic pregnancies at Ayder Comprehensive Specialized Hospital (ACSH) in Ethiopia. By identifying gaps and challenges in the current management of ectopic pregnancy, the study aims to inform policy and practice to improve maternal health services in the region.

## 2. Methods

### 2.1. Study Area, Study Period, and Study Design

This study was conducted at Ayder Comprehensive Specialized Hospital (ACSH), one of the largest tertiary hospitals in the Tigray region of Ethiopia. The hospital serves over 300,000 patients annually, primarily from the Tigray region, with a smaller proportion from neighboring districts in the Amhara and Afar regional states. Each year, it records an average of 5000 deliveries. However, ACSH does not have a dedicated obstetric ICU or high‐dependency unit, resulting in obstetric cases being admitted to the limited medical and surgical ICUs. The research employed a cross‐sectional design, covering the period from January 1, 2017 to December 31, 2021.

### 2.2. Study Population and Sample Size

This study was conducted among 152 women diagnosed with ectopic pregnancy and consecutively admitted to ACSH from 2017 to 2021.

### 2.3. Study Variables and Source of Data

Data related to sociodemographic characteristics, obstetric history, signs and symptoms, laboratory investigation results, diagnosis methods, site of ectopic pregnancy, type of ectopic pregnancy, intraoperative and postoperative management, and length of hospital stay were retrospectively collected by reviewing medical records of the 152 ectopic pregnancy cases.

### 2.4. Data Collection Procedure

As an entry point to access patient charts, the hospital’s electronic medical record system, as well as the emergency room, inpatient wards, and operating theatre registers, were consulted. Moreover, additional entries were looked into maternal near‐miss and maternal death registries. Once the entries were collected, patient charts were retrieved from the archives room. Using a structured and pretested questionnaire, data were collected by trained obstetrics and gynecology specialists. EpiData 4.6 was used as data entry software to maintain skip logic, data consistency, typing error, and ease data export to statistical software.

### 2.5. Data Analysis

Data were exported to STATA 16 for analysis. Categorical variables are described using frequency, percent with its 95% confidence interval, and graphs. Continuous variables are described using an appropriate combination of measure of central tendency and measure of dispersion.

### 2.6. Ethical Considerations

Ethical approval was obtained from the Institutional Review Board (IRB) (MU‐IRB 1950/2022) of Mekelle University, College of Health Sciences. This was part of a large maternal near‐miss and mortality research project conducted in ACSH. Permission for data collection was obtained from the Chief Clinical Director (CCD) of ACSH. Informed consent was not required as we used secondary data. Patient charts were accessed and reviewed from May 1, 2022 to June 30, 2022.

## 3. Results

### 3.1. Prevalence of Ectopic Pregnancy

During the study period from 2017 to 2021, there were a total of 23,090 deliveries at ACSH. A total of 152 cases of ectopic pregnancy were recorded, resulting in a prevalence of 6.58 per 1000 deliveries.

### 3.2. Sociodemographic Variables

The mean age of the study participants was 28 years (SD = 0.5). More than half (55.5%) belong to the age group 25 to 34 years. Almost all (96.1%) of them were from Tigray regional state of Ethiopia. Majority of women (78.3%) were from rural area (Table 1).

**Table 1:** Sociodemographic characteristics of women with ectopic pregnancy who were admitted to ACSH from 2017 to 2021 (*N* = 152).

**Variables**	**Number (%)**	**95% CI**
Region		
Tigray	146 (96.05)	91.44–98.23
Others (Afar and Amhara)	6 (3.95)	1.77–8.56
Residence		
Rural	119 (78.3)	70.97–84.2
Urban	33 (21.7)	15.8–29.03
Age category (years)		
≤ 24	35 (23.03)	16.9–30.45
25–34	89 (55.5)	50.5–66.17
35 and above	28 (18.4)	12.9–25.4

### 3.3. Obstetric History

Primigravida, multigravida, and grand multigravida accounted for 35 (23.0%), 89 (58.6%), and 28 (18.4%) cases of ectopic pregnancy, respectively. One‐third (33.5%) of women with ectopic pregnancy were nulliparous and the rest were primiparous (26.3%), multiparous (34.9%), and grand multiparous (5.3%). History of abortion was recorded in 43 (28.3%) of them. A history of ectopic pregnancy was reported in 10 (6.6%) of the cases. Fourteen (9.3%) of the women had undergone gynecologic surgery, with the most common type of gynecologic surgery being salpingectomy/salpingostomy (10/14, 71.4%). Four patients had a history of abdominal surgery. A history of STI/STD and multiple sexual partners were reported in 12 (7.9%) and 11 (7.2%) of the patients, respectively. Of the women with an ectopic pregnancy, 81 (53.2%) were aware of their current pregnancy and only 16 (10.5%) of them had an ANC contact (Table 2).

**Table 2:** Obstetric history of women with ectopic pregnancy who were admitted to ACSH from 2017 to 201 (*N* = 152).

**Variables**	**Number (%)**	**95% CI**
Gravidity		
One	35 (23.03)	16.97–30.45
2–4	89 (58.55)	50.5–66.17
5 and above	28 (18.42)	12.99–25.45
Parity		
Zero	51 (33.55)	26.45–41.49
One	40 (26.32)	19.88–33.95
2–4	53 (34.87)	27.67–42.84
5 and above	8 (5.26)	2.64–10.22
Abortion		
Yes	43 (28.29)	21.65–36.02
No	109 (71.71)	63.98–78.35
Prior history of ectopic pregnancy		
Yes	10 (6.58)	3.56–11.85
No	142 (93.42)	88.15–96.44
Gynecologic surgery		
Yes	14 (9.27)	5.54–15.1
No	137 (90.73)	84.9–94.46
Type of gynecologic surgery (*n* = 14)		
Salpingectomy	8 (57.14)	29.34–81.07
Salpingostomy	2 (14.29)	3.1–46.46
Laparotomy	2 (14.29)	3.1–46.6
Bilateral tubal ligation	1 (7.14)	0.81–41.99
MVA	1 (7.14)	0.81–41.99
Abdominal surgery		
Yes	4 (2.65)	0.99–6.9
No	147 (97.35)	93.1–99.01
Prior history of STD/STI		
Yes	12 (7.89)	4.52–13.44
No	140 (92.11)	86.56–95.48
Multiple sexual partner		
Yes	11 (7.24)	4.03–12.65
No	141 (92.76)	87.35–95.97
Contraceptive use		
Yes	29 (19.08)	13.56–26.17
No	123 (80.92)	73.83–86.44
Type of contraceptive use (*n* = 29)		
OCP	3 (10.34)	3.2–28.69
Implant	4 (13.79)	5.04–32.53
Injectables	11 (37.93)	21.82–57.24
Postpil	10 (34.48)	19.12–53.95
BTL	1 (3.45)	0.44–22.31
Knew her current pregnancy		
Yes	81 (53.19)	45.28–61.13
No	71 (46.71)	38.87–54.72
ANC contact		
Yes	16 (10.53)	6.52–16.55
No	136 (89.47)	83.45–93.48

### 3.4. Signs, Symptoms, and Laboratory Investigation Results During Admission

**Figure 1:**
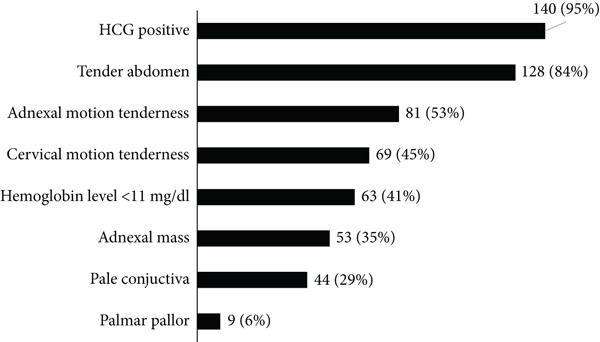
Clinical profile of women diagnosed to have ectopic pregnancy and were admitted to ACSH from 2017 to 2021 (*N* = 152).

The most common physical examination findings on arrival were tender abdomen (128, 84%), adnexal motion tenderness (81, 53%), cervical motion tenderness (69, 45%), and pale conjuctiva (44, 29%). Hemoglobin level below 11 mg/dL was detected in 63 (41%) of the cases. HCG was positive in 140 (95%) of the cases (Figure 1).

### 3.5. Diagnosis, Intraoperative Finding, and Management

Regarding the method of diagnosis, 135 (88.8%) of the cases were diagnosed both clinically and by ultrasound. The rest were diagnosed only clinically (4.6%), intraoperatively (3.3%), and only by ultrasound (3.3%). Heterotopic pregnancy was diagnosed in seven (4.6%) patients. The most common management was surgical only (92.1%), followed by medical–surgical (7.2%). Most (57.2%) of ectopic pregnancies occurred in the right tube. Ectopic pregnancies in the ovaries and in the abdomen occurred once each. Of the ectopic pregnancies that occurred in the fallopian tube, 103 (68.7%) were in the ampulla and 34 (22.7%) in the fimbriae. Pelvic adhesions were found in 59 (38.8%) of the cases. An adhered/spilled contralateral fallopian tube was found in 23 (15.1%) patients. About 80% of ectopic pregnancies were of the acute type. Salpingectomy and salpingostomy were performed in 141 (92.8%) and four (2.6%) of ectopic pregnancies, respectively. Blood transfusion was necessitated/carried out for 45 (29.6%) patients. Two patients were admitted to the intensive care unit. The median length of hospital stay was 4 days (IQR = 2) (Table 3).

**Table 3:** Diagnosis, intraoperative finding, and management of women with ectopic pregnancy who were admitted to ACSH from 2017 to 2021 (*N* = 152).

**Variables**	**Number (%)**	**95% CI**
Diagnosis method		
Clinical	7 (4.61)	1.7–2.2
Ultrasound	5 (3.29)	1.45–7.71
Clinical plus ultrasound	135 (88.82)	82.69–92.96
Intraoperative diagnosis	5 (3.29)	1.37–7.71
Heterotopic pregnancy		
Yes	7 (4.61)	2.2–9.4
No	145 (95.39)	90.6–97.8
Management		
Medical	1 (0.66)	0.09–4.49
Surgical	140 (92.11)	86.56–95.48
Medical plus surgical	11 (7.24)	4.03–12.65
Site of ectopic pregnancy		
Right tubal	87 (57.24)	49.19–64.92
Left tubal	63 (41.45)	33.83–49.5
Ovarian	1 (0.66)	0.09–4.59
Abdominal	1 (0.66)	0.09–4.59
If tubal site		
Ampulla	103 (68.67)	60.75–75.63
Isthmus	8 (5.33)	2.67–10.36
Fimbriae	34 (22.67)	16.62–30.11
Cornual	5 (3.33)	1.38–7.81
Pelvic adhesion		
Yes	59 (38.82)	31.35–46.85
No	93 (61.18)	53.15–68.65
Contralateral tube		
Healthy	129 (84.87)	78.20–89.77
Adhered/buried	23 (15.13)	10.23–21.8
Ectopic type		
Acute	121 (79.61)	72.39–85.32
Chronic	31 (20.39)	14.68–27.61
Postoperative complications		
Yes	2 (1.32)	0.33–5.19
No	149 (98.68)	94.81–99.67
Type of procedure (*N* = 151)		
Salpingectomy	140 (92.72)	87.75–96.07
Salpingostomy	4 (2.65)	0.90–6.17
Milking	5 (3.31)	1.27–7.10
Salpingoopherectomy	1 (0.66)	0.07–3.05
Omentectomy	1 (0.66)	0.07–3.05
Was she transfused		
Yes	45 (29.61)	22.84–37.4
No	107 (70.39)	62.6–77.16
Number of blood units		
One	3 (6.67)	2.10–19.24
Two	20 (44.44)	30.41–59.42
Three and above	22 (48.69)	34.4–63.57
ICU admission		
Yes	2 (1.32)	0.33–5.16
No	150 (98.68)	94.84–99.67
Length of stay in hospital		
≤7 days	138 (90.79)	84.99–94.99
7 days and above	14 (9.21)	5.51–15.01
Mean (SD)	4.22 (±0.23)	

## 4. Discussion

The primary aim of this study was to evaluate the prevalence, clinical presentation, and management outcomes of ectopic pregnancies at ACSH over 5 years. Our results show that ectopic pregnancy continues to be a significant health problem in the region with a prevalence of 6.58 (95% CI, 5.5–7.7) per 1000 deliveries. This prevalence is within the range reported in other studies from low‐income countries but is lower than the global average of 1%–2% [3].

Comparatively, a study in Nigeria reported a prevalence of 9.2 per 1000 deliveries [16], while a study in Ethiopia’s Jimma University Medical Center found a prevalence of 6.3 per 1000 deliveries [17]. The similarity in prevalence rates suggests common risk factors and challenges across these settings.

Our study found that the average age of women with ectopic pregnancy was 28 years, with the majority (55.5%) aged between 25 and 34 years. This age distribution is consistent with worldwide data indicating that ectopic pregnancies commonly occur in women in the prime reproductive period [18, 19]. The high prevalence in this age group may be attributed to increased sexual activity, higher fertility rates, and cumulative exposure to risk factors such as sexually transmitted infections (STIs) and pelvic inflammatory disease [10].

A remarkable result is that 78.3% of the patients lived in rural areas. Rural residence is associated with delayed access to health care due to factors such as limited transportation, fewer health care facilities, and lower health literacy [20, 21]. This delay can lead to more severe outcomes, such as ruptured ectopic pregnancies requiring emergency surgical interventions [22].

Regarding obstetric history, 58.6% of the women were multigravida and 28.3% had had an abortion. Previous abortions and ectopic pregnancies are known risk factors for possible tubal damage or scarring [23, 24]. The fact that 9.3% of patients had undergone previous gynecological surgery further emphasizes the role of tubal pathology in the risk of ectopic pregnancy. The low rate of ANC utilization (10.5%) is concerning. ANC contacts are crucial for the early detection of pregnancy complications [25]. Low utilization of ANC may be due to cultural beliefs, financial constraints or lack of awareness [13, 21]. Improving ANC services and encouraging early attendance could facilitate timely diagnosis and treatment of ectopic pregnancies.

Clinically, most patients presented with acute symptoms suggestive of ectopic pregnancy, such as abdominal tenderness and adnexal motion tenderness. The high incidence of anemia (hemoglobin < 11 mg/dL) in 41% of patients indicates significant blood loss likely due to tubal rupture [22]. This finding emphasizes the severity of the clinical picture and the need for immediate management. Diagnosis was made primarily on the basis of clinical assessment and ultrasound (88.8%). While ultrasound is a valuable tool, its effectiveness depends on the availability of equipment and qualified personnel [26]. In resource‐limited settings, reliance on clinical signs and symptoms remains essential [27]. The identification of heterotopic pregnancy in 4.6% of cases is noteworthy as this is a rare condition with significant diagnostic challenges [28].

Surgical treatment was the mainstay of treatment, with salpingectomy performed in 92.8% of cases. This high rate reflects the late presentation and severity of the cases [29]. In contrast, drug treatment with methotrexate is more common in stable patients in high‐income countries [30, 31]. Moreover, the need for blood transfusions in 29.6% of patients underscores the late presentation of significant number of patients, the severity of the bleeding, and the resource burden on healthcare facilities [32, 33]. Ensuring the availability of safe blood products is crucial for the management of such emergencies.

Pelvic adhesions were found in 38.8% of patients, indicating a history of pelvic infection or previous surgery [34]. Adhesions can impair fallopian tube function and increase the risk of future ectopic pregnancies [35].

### 4.1. Strengths and Limitations

A strength of this study is the comprehensive analysis of ectopic pregnancy cases over 5 years in a large tertiary care hospital, providing valuable insights into the epidemiology and management in a resource‐poor setting. However, limitations include the retrospective design, which may be subject to information bias due to incomplete medical records. In addition, results from a single institution may not be generalizable to other regions.

## 5. Conclusions

With a prevalence of 6.58 per 1000 deliveries, ectopic pregnancy remains an important health problem in the Tigray region. Most patients presented acutely and required surgical treatment. The high prevalence among women from rural areas underlines the need for improved access to reproductive health services and early detection mechanisms. Strengthening the health care infrastructure, improving the training of health care providers, and promoting awareness among the population are key strategies to address the challenges associated with ectopic pregnancy in Ethiopia. Future research should focus on prospective studies to evaluate interventions aimed at reducing the incidence of ectopic pregnancy and improving outcomes.

**Nomenclature**
ANC: antenatal care
BTL: bilateral tubal ligation
CI: confidence interval
hCG: human chorionic gonadotropin
ICU: intensive care unit
MVA: manual vacuum aspiration
OCP: oral contraceptive pills
STD/STI: sexually transmitted disease/infection

## Conflicts of Interest

The authors declare no conflicts of interest.

## Author Contributions

• Conceptualization and study design: Hale Teka, Mohammedtahir Yahya
• Data collection: Mohammedtahir Yahya, Bisrat Tesfay, Ephrem Berhe, Fanos Gebru, Awol Yemane
• Data analysis and interpretation: Hale Teka, Mohamedawel Mohamedniguss Ebrahim
• Methodology development: Bisrat Tesfay, Ephrem Berhe, Hiluf Ebuy Abraha
• Manuscript writing – original draft: Hale Teka, Mohamedawel Mohamedniguss Ebrahim, Hiluf Ebuy Abraha, Awol Yemane
• Project administration: Hale Teka
• Manuscript review and editing: All authors

## Funding

No funding was received for this manuscript.

## Data Availability

All relevant data are within the paper.

## References

[1] Barnhart K. T. , Clinical Practice. Ectopic Pregnancy, New England Journal of Medicine. (2009) 361, no. 4, 379–387, 10.1056/NEJMcp0810384, 2-s2.0-67651177703, 19625718. 19625718

[2] Creanga A. A. , Shapiro-Mendoza C. K. , Bish C. L. , Zane S. , Berg C. J. , and Callaghan W. M. , Trends in Ectopic Pregnancy Mortality in the United States: 1980-2007, Obstetrics and Gynecology. (2011) 117, no. 4, 837–843, 10.1097/AOG.0b013e3182113c10, 2-s2.0-79953194482, 21422853. 21422853

[3] Murray H. , Baakdah H. , Bardell T. , and Tulandi T. , Diagnosis and Treatment of Ectopic Pregnancy, CMAJ. (2005) 173, no. 8, 905–912, 10.1503/cmaj.050222, 2-s2.0-27144477401, 16217116. 16217116 PMC1247706

[4] Sivalingam V. N. , Duncan W. C. , Kirk E. , Shephard L. A. , and Horne A. W. , Diagnosis and Management of Ectopic Pregnancy, Journal of Family Planning and Reproductive Health Care. (2011) 37, no. 4, 231–240, 10.1136/jfprhc-2011-0073, 2-s2.0-81055147674, 21727242. 21727242 PMC3213855

[5] Kirk E. , Papageorghiou A. T. , Condous G. , Tan L. , Bora S. , and Bourne T. , The Diagnostic Effectiveness of an Initial Transvaginal Scan in Detecting Ectopic Pregnancy, Human Reproduction. (2007) 22, no. 11, 2824–2828, 10.1093/humrep/dem283, 2-s2.0-40849085237, 17855406. 17855406

[6] Crochet J. R. , Bastian L. A. , and Chireau M. V. , Does this Woman have an Ectopic Pregnancy?: The Rational Clinical Examination Systematic Review, Journal of the American Medical Association. (2013) 309, no. 16, 1722–1729, 10.1001/jama.2013.3914, 2-s2.0-84876584553, 23613077. 23613077

[7] Lipscomb G. H. , Medical Therapy for Ectopic Pregnancy, Seminars in Reproductive Medicine. (2007) 25, no. 2, 93–98, 10.1055/s-2007-970048, 2-s2.0-33947588310, 17377896. 17377896

[8] Jurkovic D. and Wilkinson H. , Diagnosis and Management of Ectopic Pregnancy, BMJ. (2011) 10, no. 342, d3397, 10.1136/bmj.d3397, 2-s2.0-84858998688, 21665933. 21665933

[9] Tuli A. G. , Goyal S. , Livingston D. , and Kurian A. S. , Ectopic Pregnancy: A Five Year Retrospective Study in a Tertiary Care Hospital, International Journal of Reproduction, Contraception, Obstetrics and Gynecology. (2017) 4, no. 5, 1400–1403, 10.18203/2320-1770.ijrcog20150718.

[10] Shaw J. L. , Dey S. K. , Critchley H. O. , and Horne A. W. , Current Knowledge of the Aetiology of Human Tubal Ectopic Pregnancy, Human Reproduction Update. (2010) 16, no. 4, 432–444, 10.1093/humupd/dmp057, 2-s2.0-77954846935, 20071358. 20071358 PMC2880914

[11] Ehsan N. and Mehmood A. , Ectopic Pregnancy: An Analysis of 62 Cases, Journal of the Pakistan Medical Association. (1998) 48, no. 2, 26–29, 9610087. 9610087

[12] Berhan Y. and Berhan A. , Causes of Maternal Mortality in Ethiopia: A Significant Decline in Abortion Related Death, Ethiopian Journal of Health Sciences. (2014) 24, 15–28, 10.4314/ejhs.v24i0.3S, 2-s2.0-84964313590. PMC4249203 25489180

[13] Hadaro T. S. , Shewangizaw M. , Geltore T. E. , Bekele M. , Zewude L. D. , Abebe M. , Chisha Y. , Mare K. U. , and Leyto S. M. , Delays in the Decision to Seek Care and Associated Factors among Women in Ethiopia: Systematic Review and Meta-Analysis, BMC Pregnancy and Childbirth. (2025) 25, no. 1, 10.1186/s12884-025-07968-4, 40790472. PMC12341225 40790472

[14] Berhe E. T. , Kiros K. , Hagos M. G. , Gesesew H. A. , Ward P. R. , and Gebremeskel T. G. , Ectopic Pregnancy in Tigray, Ethiopia: A Cross-Sectional Survey of Prevalence, Management Outcomes, and Associated Factors, Journal of Pregnancy. (2021) 2021, 4443117, 10.1155/2021/4443117, 34888104. 34888104 PMC8651379

[15] Chudasama T. J. , Shah S. R. , Vyas R. C. , and Parikh P. M. , A Retrospective Analysis of Ectopic Pregnancies in Tertiary Care Hospital of Western India: Two Year Study, International Journal of Reproduction, Contraception, Obstetrics and Gynecology. (2020) 9, no. 8, 3336–3340, 10.18203/2320-1770.ijrcog20203319.

[16] Omokanye L. O. , Balogun O. R. , Salaudeen A. G. , Olatinwo A. W. , and Saidu R. , Ectopic Pregnancy in Ilorin, Nigeria: A Four Year Review, Nigerian Postgraduate Medical Journal. (2013) 20, no. 4, 341–345, 10.1155/2022/1491419, 24633280. 24633280

[17] Gerema U. , Alemayehu T. , Chane G. , Desta D. , and Diriba A. , Determinants of Ectopic Pregnancy among Pregnant Women Attending Referral Hospitals in Southwestern Part of Oromia Regional State, Southwest Ethiopia: a Multi-Center Case Control Study, BMC Pregnancy and Childbirth. (2021) 21, no. 1, 10.1186/s12884-021-03618-7, 33579224. PMC7881641 33579224

[18] Mbalinda S. N. , Nakimuli A. , Kakaire O. , Osinde M. O. , Kakande N. , and Kaye D. K. , Does Knowledge of Danger Signs of Pregnancy Predict Birth Preparedness? A Critique of the Evidence from Women Admitted with Pregnancy Complications, Health Research Policy and Systems. (2014) 12, 10.1186/1478-4505-12-60, 2-s2.0-84907953355, 25300499. PMC4197291 25300499

[19] Bouyer J. , Job-Spira N. , Pouly J. L. , Coste J. , Germain E. , and Fernandez H. , Fertility following Radical, Conservative-Surgical or Medical Treatment for Tubal Pregnancy: A Population-Based Study, BJOG : An International Journal of Obstetrics and Gynaecology. (2000) 107, no. 6, 714–721, 10.1111/j.1471-0528.2000.tb13330.x, 2-s2.0-0034065389, 10847225. 10847225

[20] Gebrekidan K. and Worku A. , Factors Associated with Late ANC Initiation among Pregnant Women in Select Public Health Centers of Addis Ababa, Ethiopia: Unmatched Case-Control Study Design, Pragmatic and Observational Research. (2017) 26, no. 8, 223–230, 10.2147/POR.S140733, 29138615. PMC5667792 29138615

[21] Yemane G. D. , The Factors Associated with Antenatal Care Utilization in Ethiopia, Annals of Medicine and Surgery. (2022) 30, no. 79, 104092, 10.1016/j.amsu.2022.104092, 35860111. PMC9289506 35860111

[22] Panti A. , Ikechukwu N. E. , Lukman O. O. , Yakubu A. , Egondu S. C. , and Tanko B. A. , Ectopic Pregnancy at Usmanu Danfodiyo University Teaching Hospital Sokoto: A Ten year Review, Annals of Nigerian Medicine. (2012) 6, no. 2, 87–91, 10.4103/0331-3131.108128.

[23] Bouyer J. , Coste J. , Shojaei T. , Pouly J. L. , Fernandez H. , Gerbaud L. , and Job-Spira N. , Risk Factors for Ectopic Pregnancy: A Comprehensive Analysis Based on a Large Case-Control, Population-Based Study in France, American Journal of Epidemiology. (2003) 157, no. 3, 185–194, 10.1093/aje/kwf190, 2-s2.0-0037308060, 12543617. 12543617

[24] Bouyer J. , Coste J. , Fernandez H. , Pouly J. L. , and Job-Spira N. , Sites of Ectopic Pregnancy: A 10 year Population-Based Study of 1800 Cases, Human Reproduction. (2002) 17, no. 12, 3224–3230, 10.1093/humrep/17.12.3224, 12456628. 12456628

[25] World Health Organization (WHO) , Medical Management of Abortion, 2018, World Health Organisation, https://www.who.int/publications/i/item/9789241549912.

[26] Casikar I. , Reid S. , and Condous G. , Ectopic Pregnancy: Ultrasound Diagnosis in Modern Management, Clinical Obstetrics and Gynecology. (2012) 55, no. 2, 402–409, 10.1097/GRF.0b013e31825109bd, 2-s2.0-84860283143, 22510621. 22510621

[27] Lozeau A. M. and Potter B. , Diagnosis and Management of Ectopic Pregnancy, American Family Physician. (2005) 72, no. 9, 1707–1714, Erratum in: *American Family Physician* 2007;75(3):31216300032. 16300032

[28] Li C. , Zhao W. H. , Zhu Q. , Cao S. J. , Ping H. , Xi X. , Qin G. J. , Yan M. X. , Zhang D. , Qiu J. , and Zhang J. , Risk Factors for Ectopic Pregnancy: A Multi-Center Case-Control Study, BMC Pregnancy and Childbirth. (2015) 15, 10.1186/s12884-015-0613-1, 2-s2.0-84939523503. PMC4546260 26296545

[29] Majhi A. K. , Roy N. , Karmakar K. S. , and Banerjee P. K. , Ectopic Pregnancy—An Analysis of 180 Cases, Journal of the Indian Medical Association. (2007) 105, no. 6, 308–310, 18232175. 18232175

[30] Practice Committee of American Society for Reproductive Medicine , Medical Treatment of Ectopic Pregnancy: A Committee Opinion, Fertility and Sterility. (2013) 100, no. 3, 638–644, 10.1016/j.fertnstert.2013.06.013, 2-s2.0-84883444909, 23849842. 23849842

[31] Lipscomb G. H. , Bran D. , McCord M. L. , Portera J. C. , and Ling F. W. , Analysis of Three Hundred Fifteen Ectopic Pregnancies Treated with Single-Dose Methotrexate, American Journal of Obstetrics and Gynecology. (1998) 178, no. 6, 1354–1358, 10.1016/s0002-9378(98)70343-6, 2-s2.0-0031777152, 9662322. 9662322

[32] Gizaw N. T. , K/Mariam M. A. , and Fayera M. G. , Magnitude of Ectopic Pregnancy, Management Methods, and its Associated Factors among Pregnant Women attending Ambo University Referral Hospital in Oromia Regional State, Ethiopia: A Seven years Retrospective Institutional based Cross-Sectional Study, PLOS Glob Public Health. (2025) 5, no. 6, e0004611, 10.1371/journal.pgph.0004611, 40471945. 40471945 PMC12140202

[33] Cullifer R. , Johnson C. , Huynh T. , Yeonjoo Y. , Pacis M. , Hoffman M. , Swartz S. , and Makai G. , Preoperative risk factors for blood transfusion in women requiring surgical management of ectopic pregnancy: a retrospective cohort study, Gynecology and Obstetrics Clinical Medicine. (2024) 4, e000057, 10.1136/gocm-2024-000057.

[34] Al-Sunaidi M. and Tulandi T. , Surgical treatment of ectopic pregnancy, Seminars in Reproductive Medicine. (2007) 25, no. 2, 117–122, 10.1055/s-2007-970050, 2-s2.0-33947584455, 17377898. 17377898

[35] Mol F. , van Mello N. M. , Strandell A. , Strandell K. , Jurkovic D. , Ross J. , Barnhart K. T. , Yalcinkaya T. M. , Verhoeve H. R. , Graziosi G. C. M. , Koks C. A. M. , Klinte I. , Hogström L. , Janssen I. C. A. H. , Kragt H. , Hoek A. , Trimbos-Kemper T. C. M. , Broekmans F. J. M. , Willemsen W. N. P. , Ankum W. M. , Mol B. W. , van Wely M. , van der Veen F. , and Hajenius P. J. , European Surgery in Ectopic Pregnancy (ESEP) Study Group. Salpingotomy versus Salpingectomy in Women with Tubal Pregnancy (ESEP Study): An Open-Label, Multicentre, Randomised Controlled Trial, Lancet. (2014) 383, no. 9927, 1483–1489, 10.1016/S0140-6736(14)60123-9, 2-s2.0-84899635148, 24499812. 24499812

